# Supplementary material for: Molecular Manipulation of the miR399/PHO2 Expression Module Alters the Salt Stress Response of Arabidopsis thaliana
Source: Plants (Basel). 2020 Dec 31;10(1):73. doi: 10.3390/plants10010073 (PMC7824465; doi:10.3390/plants10010073)
Supplement: Supplementary file 1 [file plants-10-00073-s001.pdf]

Supplementary Table 1: Oligonucleotide sequence of DNA primers used in this study. Primers listed were used for miR399-specific cDNA synthesis, RT-qPCR assessment of mRNA transcript abundance or SL-RT-qPCR assessment of miR399 accumulation.

Transcript	Gene ID	Oligonucleotide sequence (5' to 3')
P5CS1	AT2G39800	**FP:** GTTTTTGAATCCCGACCTGA
		**RP:** TTACCCCCAACAGTCTCTGG
PHO2	AT2G33770	**FP:** ACCGTTTCTCATCAAGGCGT
		**RP:** GTGCCCGTCCACCATAAGAA
PHR1	AT4G28610	**FP:** AAACCAACCCGGCGATTCA
		**RP:** CAGCCCATTCATGCCAATCACTT
PHT1;4	AT2G38940	**FP:** TGTGCCGGCCGAAATCT
		**RP:** TTGCTCCTAATTTTCCTGATGCT
PHT1;9	AT1G76430	**FP:** TGGAGCTGCAGGGAAGTTTG
		**RP:** ATCTGGAAAACCGTCCTCTTCAT
UBI10	AT4G05320	**FP:** GGCCTTGTATAATCCCTGATGAATAAG
		**RP:** AAAGAGATAACAGGAACGGAAACATA
Universal-SLR		**RP:** CCAGTGCAGGGTCCGAGGTA
snoR101		**FP:** CTTCACAGGTAAGTTCGCTTG
		**RP:** AGCATCAGCAGACCAGTAGTT
miR399		**FP:** AACGTGCCTGGCTCCCTGTATGCC
		**RTSL:** GTCGTATCCAGTGCAGGGTCCGAGGTATTCGCACTGGATACGACTGGCAT
